# *N*-(Benzothia­zol-2-yl)-3-chloro­benzamide

**DOI:** 10.1107/S1600536809016481

**Published:** 2009-05-14

**Authors:** M. Khawar Rauf, Michael Bolte, Amin Badshah

**Affiliations:** a Department of Chemistry, Quaid-i-Azam University, Islamabad 45320, Pakistan; b Institut für Anorganische Chemie, J. W. Goethe-Universität Frankfurt, Max-von-Laue-Strasse 7, 60438 Frankfurt/Main, Germany; c Department of Chemistry, Islamia University of Bahawalpur, Pakistan

## Abstract

The title mol­ecule, C_14_H_9_ClN_2_OS, exists in the solid state in its amide form with a typical C=O bond length, as well as shortened C—N bonds. The plane containing the HNCO atoms subtends dihedral angles of 12.3 (4) and 8.1 (3)° with the planes of the phenyl ring and benzothia­zole group, respectively, whereas the dihedral angle between the planes of the phenyl ring and the benzothia­zole group is 5.96 (6)°. In the crystal, mol­ecules form inter­molecular N—H⋯N hydrogen bonds, generating independent scissor-like *R* ^2^_2_(8) dimers.

## Related literature

For geometric data, see: Allen *et al.* (1987 ▶); For related structures, see: Garden *et al.* (2005 ▶); Wardell *et al.* (2005 ▶).
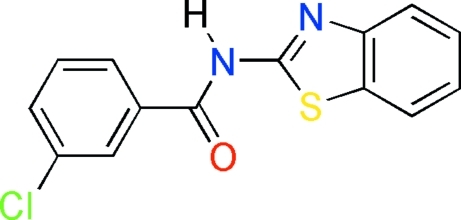


## Experimental

### 

#### Crystal data

C_14_H_9_ClN_2_OS
*M* *_r_* = 288.74
Monoclinic, 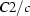

*a* = 26.6613 (19) Å
*b* = 7.5766 (5) Å
*c* = 12.6729 (10) Å
β = 99.729 (6)°
*V* = 2523.1 (3) Å^3^
*Z* = 8
Mo *K*α radiation
μ = 0.46 mm^−1^
*T* = 173 K
0.39 × 0.38 × 0.35 mm

#### Data collection

Stoe IPDS II two-circle diffractometer
Absorption correction: multi-scan [*MULABS* (Spek, 2009 ▶); Blessing, 1995 ▶)] *T*_min_ = 0.841, *T*_max_ = 0.856
9132 measured reflections
2352 independent reflections
2084 reflections with *I* > 2σ(*I*)
*R*_int_ = 0.038

#### Refinement

*R*[*F* ^2^ > 2σ(*F* ^2^)] = 0.028
*wR*(*F* ^2^) = 0.079
*S* = 1.03
2352 reflections
177 parameters
H atoms treated by a mixture of independent and constrained refinement
Δρ_max_ = 0.29 e Å^−3^
Δρ_min_ = −0.23 e Å^−3^

### 

Data collection: *X-AREA* (Stoe & Cie, 2001 ▶); cell refinement: *X-AREA*; data reduction: *X-AREA*; program(s) used to solve structure: *SHELXS97* (Sheldrick, 2008 ▶); program(s) used to refine structure: *SHELXL97* (Sheldrick, 2008 ▶); molecular graphics: *PLATON* (Spek, 2009 ▶) and *XP* in *SHELXTL-Plus* (Sheldrick, 2008 ▶); software used to prepare material for publication: *SHELXL97*.

## Supplementary Material

Crystal structure: contains datablocks I, global. DOI: 10.1107/S1600536809016481/hg2508sup1.cif

Structure factors: contains datablocks I. DOI: 10.1107/S1600536809016481/hg2508Isup2.hkl

Additional supplementary materials:  crystallographic information; 3D view; checkCIF report

## Figures and Tables

**Table 1:** Hydrogen-bond geometry (Å, °)

*D*—H⋯*A*	*D*—H	H⋯*A*	*D*⋯*A*	*D*—H⋯*A*
N1—H1⋯N2^i^	0.94 (2)	2.02 (2)	2.9429 (18)	168.9 (17)

Symmetry code: (i) 
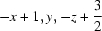
.

**Fig. 1.**
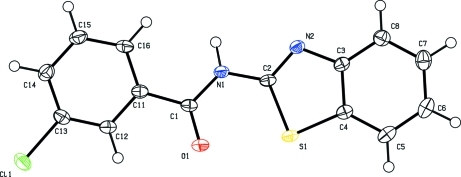
Molecular structure of (I) showing atom-numbering scheme. Displacement ellipsoids are drawn at the 50% probability level.

**Fig. 2.**
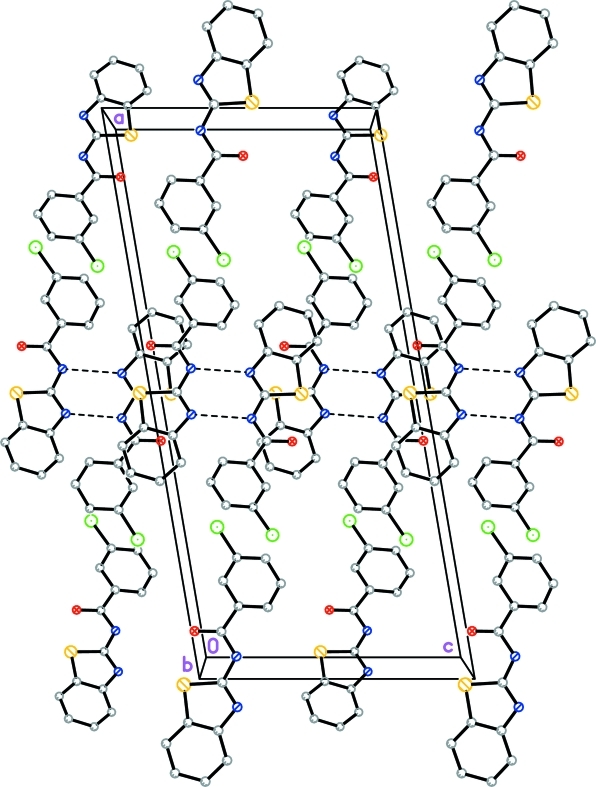
Packing diagram of (I) with view onto the ac plane. Hydrogen bonds shown as dashed lines. H atoms are omitted for clarity.

## supplementary crystallographic information

### Comment

We report here the structure of the title compound, (I) (Fig. 1), which has been separated from an impure sample of thiourea by column chromatography as a by-product, a part of our ongoing studies related to *N*,*N'*-disubstituted thioureas and heterocyclic compounds. These include N—H···N hydrogen bonds, with possible oxygen-sulfur intramolecular interactions (Fig. 2). In this class of componds, N—H···O, C—H···O and N—H···N hydrogen bonds, and weak π–π stacking interactions are the only direction-specific intermolecular interactions (Garden *et al.*, 2005; Wardell *et al.*, 2005). The molecules of (I) are nearly planar, as shown by the leading torsion angles [C11—C1—N1—C2 174.90 (12) and C1—N1—C2—N2 -171.57 (13)°], and the amide group adopts the usual *trans* conformation; the bond lengths and inter-bond angles present no unusual values (Allen *et al.*, 1987).

### Experimental

Freshly prepared and steam-distilled 3-chlorobenzoyl isothiocyanate (1.98 g, 10 mmol) was stirred in acetone (30 ml) for 20 min. Neat 2-aminobenzothiazole (1.50 g, 10 mmol) was then added and the resulting mixture was stirred for 1 h. The reaction mixture was then poured into 300 ml (approx.) acidified (pH 4) water and stirred well. The solid product was separated and washed with deionized water. One of the fraction obtained as a by-product during the column chromatography of the target thiourea was recrystallized from methanol/1,1-dichloromethane (1:10 *v*/*v*) to give fine crystals of (I), with an overall fractional yield of 15%.

### Refinement

H atoms bonded to C were included in calculated positions and refined as riding on their parent C atom with C—H = 0.95 Å and *U*_iso_(H) = 1.2*U*_eq_(C). The H atom bonded to N was freely refined.

### Crystal data

**Table:**

C_14_H_9_ClN_2_OS	*F*(000) = 1184
*M**_r_* = 288.74	*D*_x_ = 1.520 Mg m^−^^3^
Monoclinic, *C*2/*c*	Mo *K*α radiation, λ = 0.71073 Å
Hall symbol: -C 2yc	Cell parameters from 8429 reflections
*a* = 26.6613 (19) Å	θ = 3.6–25.9°
*b* = 7.5766 (5) Å	µ = 0.46 mm^−^^1^
*c* = 12.6729 (10) Å	*T* = 173 K
β = 99.729 (6)°	Block, light yellow
*V* = 2523.1 (3) Å^3^	0.39 × 0.38 × 0.35 mm
*Z* = 8	

### Data collection

**Table:**

Stoe IPDS II two-circle diffractometer	2352 independent reflections
Radiation source: fine-focus sealed tube	2084 reflections with *I* > 2σ(*I*)
graphite	*R*_int_ = 0.038
ω scans	θ_max_ = 25.6°, θ_min_ = 3.6°
Absorption correction: multi-scan [*MULABS* (Spek, 2009); Blessing, 1995)]	*h* = −32→32
*T*_min_ = 0.841, *T*_max_ = 0.856	*k* = −8→9
9132 measured reflections	*l* = −15→15

### Refinement

**Table:**

Refinement on *F*^2^	Secondary atom site location: difference Fourier map
Least-squares matrix: full	Hydrogen site location: inferred from neighbouring sites
*R*[*F*^2^ > 2σ(*F*^2^)] = 0.028	H atoms treated by a mixture of independent and constrained refinement
*wR*(*F*^2^) = 0.079	*w* = 1/[σ^2^(*F*_o_^2^) + (0.051*P*)^2^ + 0.8598*P*] where *P* = (*F*_o_^2^ + 2*F*_c_^2^)/3
*S* = 1.03	(Δ/σ)_max_ = 0.001
2352 reflections	Δρ_max_ = 0.28 e Å^−^^3^
177 parameters	Δρ_min_ = −0.22 e Å^−^^3^
0 restraints	Extinction correction: *SHELXL97* (Sheldrick, 2008), Fc^*^=kFc[1+0.001xFc^2^λ^3^/sin(2θ)]^-1/4^
Primary atom site location: structure-invariant direct methods	Extinction coefficient: 0.0071 (6)

### Special details

**Table:**

Geometry. All e.s.d.'s (except the e.s.d. in the dihedral angle between two l.s. planes) are estimated using the full covariance matrix. The cell e.s.d.'s are taken into account individually in the estimation of e.s.d.'s in distances, angles and torsion angles; correlations between e.s.d.'s in cell parameters are only used when they are defined by crystal symmetry. An approximate (isotropic) treatment of cell e.s.d.'s is used for estimating e.s.d.'s involving l.s. planes.
Refinement. Refinement of *F*^2^ against ALL reflections. The weighted *R*-factor *wR* and goodness of fit *S* are based on *F*^2^, conventional *R*-factors *R* are based on *F*, with *F* set to zero for negative *F*^2^. The threshold expression of *F*^2^ > σ(*F*^2^) is used only for calculating *R*-factors(gt) *etc*. and is not relevant to the choice of reflections for refinement. *R*-factors based on *F*^2^ are statistically about twice as large as those based on *F*, and *R*- factors based on ALL data will be even larger.

### Fractional atomic coordinates and isotropic or equivalent isotropic displacement parameters (Å^2^)

**Table:**

	*x*	*y*	*z*	*U*_iso_*/*U*_eq_	
Cl1	0.762196 (14)	0.69837 (6)	0.65336 (3)	0.03053 (14)	
S1	0.500738 (13)	0.23662 (5)	0.45176 (3)	0.01915 (13)	
O1	0.58693 (4)	0.40431 (15)	0.51591 (8)	0.0241 (3)	
N1	0.54529 (4)	0.34541 (16)	0.65241 (10)	0.0178 (3)	
H1	0.5448 (7)	0.325 (2)	0.7252 (17)	0.032 (5)*	
C1	0.58602 (5)	0.41061 (19)	0.61190 (11)	0.0179 (3)	
N2	0.46256 (4)	0.23590 (16)	0.62817 (10)	0.0185 (3)	
C2	0.50288 (5)	0.27653 (19)	0.58813 (11)	0.0167 (3)	
C3	0.42583 (5)	0.16277 (19)	0.54925 (12)	0.0185 (3)	
C4	0.44015 (5)	0.15038 (19)	0.44819 (12)	0.0191 (3)	
C5	0.40794 (6)	0.0750 (2)	0.36146 (12)	0.0242 (3)	
H5	0.4179	0.0661	0.2932	0.029*	
C6	0.36121 (6)	0.0141 (2)	0.37817 (13)	0.0275 (4)	
H6	0.3388	−0.0391	0.3207	0.033*	
C7	0.34626 (6)	0.0292 (2)	0.47829 (13)	0.0273 (4)	
H7	0.3136	−0.0118	0.4873	0.033*	
C8	0.37807 (5)	0.1024 (2)	0.56412 (12)	0.0234 (3)	
H8	0.3677	0.1117	0.6320	0.028*	
C11	0.62782 (5)	0.49568 (19)	0.68791 (11)	0.0188 (3)	
C12	0.67079 (5)	0.5438 (2)	0.64573 (12)	0.0200 (3)	
H12	0.6734	0.5140	0.5740	0.024*	
C13	0.70955 (5)	0.6348 (2)	0.70855 (12)	0.0213 (3)	
C14	0.70650 (6)	0.6824 (2)	0.81238 (12)	0.0245 (3)	
H14	0.7333	0.7463	0.8547	0.029*	
C15	0.66341 (6)	0.6349 (2)	0.85358 (12)	0.0249 (3)	
H15	0.6607	0.6672	0.9248	0.030*	
C16	0.62434 (6)	0.5413 (2)	0.79260 (12)	0.0220 (3)	
H16	0.5953	0.5083	0.8222	0.026*	

### Atomic displacement parameters (Å^2^)

**Table:**

	*U*^11^	*U*^22^	*U*^33^	*U*^12^	*U*^13^	*U*^23^
Cl1	0.0200 (2)	0.0423 (3)	0.0307 (2)	−0.00460 (15)	0.00835 (16)	0.00331 (17)
S1	0.0228 (2)	0.0232 (2)	0.0121 (2)	0.00280 (14)	0.00466 (14)	0.00001 (13)
O1	0.0263 (6)	0.0325 (6)	0.0148 (6)	−0.0019 (4)	0.0074 (4)	−0.0004 (4)
N1	0.0188 (6)	0.0221 (7)	0.0128 (6)	0.0014 (5)	0.0041 (5)	0.0011 (5)
C1	0.0199 (7)	0.0189 (7)	0.0161 (8)	0.0046 (5)	0.0061 (5)	0.0018 (5)
N2	0.0183 (6)	0.0227 (7)	0.0143 (6)	0.0024 (5)	0.0019 (5)	0.0000 (5)
C2	0.0207 (7)	0.0178 (7)	0.0121 (7)	0.0050 (5)	0.0040 (5)	0.0019 (5)
C3	0.0203 (7)	0.0188 (7)	0.0156 (7)	0.0042 (6)	0.0008 (5)	0.0014 (5)
C4	0.0220 (7)	0.0184 (7)	0.0167 (7)	0.0044 (6)	0.0024 (6)	0.0011 (6)
C5	0.0322 (8)	0.0214 (8)	0.0172 (8)	0.0050 (6)	−0.0009 (6)	−0.0020 (6)
C6	0.0301 (8)	0.0230 (8)	0.0256 (9)	0.0001 (6)	−0.0060 (6)	−0.0029 (6)
C7	0.0233 (8)	0.0253 (9)	0.0311 (9)	−0.0005 (6)	−0.0015 (6)	0.0036 (7)
C8	0.0225 (7)	0.0260 (9)	0.0215 (8)	0.0026 (6)	0.0030 (6)	0.0032 (6)
C11	0.0220 (7)	0.0182 (7)	0.0170 (7)	0.0033 (6)	0.0053 (6)	0.0021 (6)
C12	0.0220 (7)	0.0228 (8)	0.0162 (7)	0.0034 (6)	0.0061 (6)	0.0008 (6)
C13	0.0198 (7)	0.0231 (8)	0.0221 (8)	0.0012 (6)	0.0067 (6)	0.0048 (6)
C14	0.0280 (8)	0.0240 (8)	0.0202 (8)	−0.0027 (6)	0.0004 (6)	0.0010 (6)
C15	0.0337 (8)	0.0259 (8)	0.0159 (7)	−0.0028 (7)	0.0063 (6)	0.0000 (6)
C16	0.0267 (8)	0.0241 (8)	0.0173 (8)	−0.0004 (6)	0.0095 (6)	0.0027 (6)

### Geometric parameters (Å, °)

**Table:**

Cl1—C13	1.7386 (15)	C6—C7	1.397 (2)
S1—C4	1.7360 (15)	C6—H6	0.9500
S1—C2	1.7459 (14)	C7—C8	1.378 (2)
O1—C1	1.2216 (18)	C7—H7	0.9500
N1—C1	1.3696 (18)	C8—H8	0.9500
N1—C2	1.3794 (18)	C11—C16	1.389 (2)
N1—H1	0.94 (2)	C11—C12	1.392 (2)
C1—C11	1.491 (2)	C12—C13	1.378 (2)
N2—C2	1.3008 (19)	C12—H12	0.9500
N2—C3	1.3912 (18)	C13—C14	1.380 (2)
C3—C8	1.395 (2)	C14—C15	1.388 (2)
C3—C4	1.400 (2)	C14—H14	0.9500
C4—C5	1.397 (2)	C15—C16	1.383 (2)
C5—C6	1.378 (2)	C15—H15	0.9500
C5—H5	0.9500	C16—H16	0.9500
			
C4—S1—C2	88.03 (7)	C8—C7—H7	119.4
C1—N1—C2	122.53 (12)	C6—C7—H7	119.4
C1—N1—H1	125.0 (11)	C7—C8—C3	118.64 (14)
C2—N1—H1	111.8 (11)	C7—C8—H8	120.7
O1—C1—N1	120.63 (13)	C3—C8—H8	120.7
O1—C1—C11	121.46 (13)	C16—C11—C12	119.59 (14)
N1—C1—C11	117.86 (12)	C16—C11—C1	124.15 (13)
C2—N2—C3	109.98 (12)	C12—C11—C1	116.03 (12)
N2—C2—N1	120.53 (13)	C13—C12—C11	119.64 (13)
N2—C2—S1	117.01 (11)	C13—C12—H12	120.2
N1—C2—S1	122.45 (11)	C11—C12—H12	120.2
N2—C3—C8	125.53 (13)	C12—C13—C14	121.49 (14)
N2—C3—C4	114.62 (13)	C12—C13—Cl1	118.93 (12)
C8—C3—C4	119.85 (14)	C14—C13—Cl1	119.52 (12)
C5—C4—C3	121.38 (14)	C13—C14—C15	118.48 (14)
C5—C4—S1	128.30 (12)	C13—C14—H14	120.8
C3—C4—S1	110.32 (11)	C15—C14—H14	120.8
C6—C5—C4	117.87 (15)	C16—C15—C14	121.08 (14)
C6—C5—H5	121.1	C16—C15—H15	119.5
C4—C5—H5	121.1	C14—C15—H15	119.5
C5—C6—C7	121.06 (14)	C15—C16—C11	119.71 (13)
C5—C6—H6	119.5	C15—C16—H16	120.1
C7—C6—H6	119.5	C11—C16—H16	120.1
C8—C7—C6	121.19 (15)		
			
C2—N1—C1—O1	−2.6 (2)	C5—C6—C7—C8	−1.2 (2)
C2—N1—C1—C11	174.90 (12)	C6—C7—C8—C3	0.3 (2)
C3—N2—C2—N1	−177.62 (12)	N2—C3—C8—C7	−178.45 (14)
C3—N2—C2—S1	1.43 (16)	C4—C3—C8—C7	0.9 (2)
C1—N1—C2—N2	−171.57 (13)	O1—C1—C11—C16	165.51 (14)
C1—N1—C2—S1	9.43 (19)	N1—C1—C11—C16	−11.9 (2)
C4—S1—C2—N2	−1.80 (12)	O1—C1—C11—C12	−8.9 (2)
C4—S1—C2—N1	177.23 (12)	N1—C1—C11—C12	173.61 (12)
C2—N2—C3—C8	179.29 (14)	C16—C11—C12—C13	0.5 (2)
C2—N2—C3—C4	−0.12 (18)	C1—C11—C12—C13	175.22 (13)
N2—C3—C4—C5	178.08 (13)	C11—C12—C13—C14	−1.1 (2)
C8—C3—C4—C5	−1.4 (2)	C11—C12—C13—Cl1	−178.45 (11)
N2—C3—C4—S1	−1.19 (16)	C12—C13—C14—C15	0.7 (2)
C8—C3—C4—S1	179.37 (11)	Cl1—C13—C14—C15	178.04 (12)
C2—S1—C4—C5	−177.64 (15)	C13—C14—C15—C16	0.3 (2)
C2—S1—C4—C3	1.56 (11)	C14—C15—C16—C11	−0.8 (2)
C3—C4—C5—C6	0.5 (2)	C12—C11—C16—C15	0.4 (2)
S1—C4—C5—C6	179.62 (12)	C1—C11—C16—C15	−173.83 (14)
C4—C5—C6—C7	0.8 (2)		

### Hydrogen-bond geometry (Å, °)

**Table:**

*D*—H···*A*	*D*—H	H···*A*	*D*···*A*	*D*—H···*A*
N1—H1···N2^i^	0.94 (2)	2.02 (2)	2.9429 (18)	168.9 (17)

Symmetry codes: (i) −*x*+1, *y*, −*z*+3/2.
